# Supplementary material for: Microplastic-Mediated Transfer of Tetracycline Resistance: Unveiling the Role of Mussels in Marine Ecosystems
Source: Antibiotics (Basel). 2024 Aug 2;13(8):727. doi: 10.3390/antibiotics13080727 (PMC11350897; doi:10.3390/antibiotics13080727)
Supplement: Supplementary file 1 [file antibiotics-13-00727-s001.zip › antibiotics-3132756-supplementary.pdf]

Table S1. Results of conjugation events occurring in experimental condition II. The table shows the donor (D) and transconjugant (T) concentrations (CFU/ml) and *TetM* transfer rate (Log T/D) obtained by sampling the aquarium water for both recipient strains. All experiments were conducted in triplicates (A, B, C).

		4 Days			7 Days		
**Recipient**	**Sample**	**Donor (CFU/ml)**	**Transconjugant (CFU/ml)**	**Log (T/D)**	**Donor (CFU/ml)**	**Transconjugant (CFU/ml)**	**Log (T/D)**
L. monocytogenes DSM 15675	Water A	9,50E+08	1,60E+01	-7,77	7,50E+08	4,60E+02	-6,21
	Water B	6,70E+08	8,00E+00	-7,92	9,50E+08	2,60E+02	-6,56
	Water C	6,30E+08	6,00E+00	-8,02	7,00E+08	2,40E+02	-6,46
L. monocytogenes ScottA	Water A	7,00E+08	1,20E+01	-7,77	5,50E+08	2,10E+02	-6,42
	Water B	9,00E+08	8,00E+00	-8,05	5,50E+08	3,80E+02	-6,16
	Water C	9,50E+08	1,00E+01	-7,98	7,00E+08	3,70E+02	-6,28

Table S2. Results of conjugation occurring in experimental condition IV. The table shows the donor and transconjugant concentrations (CFU/ml) and *TetM* transfer rate (Log T/D) obtained by sampling the aquarium water and *M. galloprovincialis* for both recipient strains. All experiments were conducted in triplicates (A, B, C).

		4 Days			7 Days		
**Recipient**	**Sample**	**Donor (CFU/ml)**	**Transconjugant (CFU/ml)**	**Log (T/D)**	**Donor (CFU/ml)**	**Transconjugant (CFU/ml)**	**Log (T/D)**
	Water A	5,30E+07	6,00E+00	-6,95	3,70E+07	9,00E+00	-6,61

L. monocytogenes DSM 15675	Water B	5,00E+07	7,00E+00	-6,85	2,90E+07	8,00E+00	-6,56
	Water C	6,70E+07	8,00E+00	-6,92	3,50E+07	8,00E+00	-6,64
	M. galloprovincialis A	8,70E+07	2,40E+02	-5,56	4,40E+08	3,00E+03	-5,17
	M. galloprovincialis B	7,70E+07	2,80E+02	-5,44	4,40E+08	3,40E+03	-5,11
	M. galloprovincialis C	6,80E+07	2,50E+02	-5,43	5,50E+08	3,20E+03	-5,24
L. monocytogenes ScottA	Water A	5,30E+07	8,00E+00	-6,82	3,30E+07	9,00E+00	-6,56
	Water B	4,80E+07	8,00E+00	-6,78	4,80E+07	8,00E+00	-6,78
	Water C	5,50E+07	7,00E+00	-6,90	3,50E+07	7,00E+00	-6,70
	M. galloprovincialis A	7,80E+07	2,40E+02	-5,51	4,50E+08	4,20E+03	-5,03
	M. galloprovincialis B	7,80E+07	2,50E+02	-5,49	4,80E+08	4,20E+03	-5,06
	M. galloprovincialis C	7,90E+07	2,70E+02	-5,47	5,00E+08	4,10E+03	-5,09

Table S3. Overview of the results for the conjugation frequencies and relative standard deviation obtained under the different experimental conditions. The results are expressed as the mean of three independent replicates.

Recipient	Experimental condition	Sample type	Conjugation Frequency [Log(T/D)]	SD	Conjugation Frequency [Log(T/D)]	SD
L. monocytogenes DSM 15675	***I***	**Water**	n.d. *	n.d.	n.d.	n.d.
	II	**Water**	-7,91	0,12	-6,41	0,18
	III	***M. galloprovincialis***	n.d.	n.d.	n.d.	n.d.
		**Water**	n.d.	n.d.	n.d.	n.d.

	IV	***M. galloprovincialis***	-5,48	0,07	-5,17	0,06
		**Water**	-6,91	0,05	-6,60	0,04
L. monocytogenes ScottA	***I***	**Water**	n.d.	n.d.	n.d.	n.d.
	II	**Water**	-7,93	0,15	-6,29	0,13
	III	***M. galloprovincialis***	n.d.	n.d.	n.d.	n.d.
		**Water**	n.d.	n.d.	n.d.	n.d.
	IV	***M. galloprovincialis***	-5,49	0,02	-5,06	0,03
		**Water**	-6,83	0,06	-6,68	0,11

* N.d. not determined
